# Effect of lentivirus-mediated shRNA inactivation of HK1, HK2, and HK3 genes in colorectal cancer and melanoma cells

**DOI:** 10.1186/s12863-016-0459-1

**Published:** 2016-12-22

**Authors:** Anna V. Kudryavtseva, Maria S. Fedorova, Alex Zhavoronkov, Alexey A. Moskalev, Alexander S. Zasedatelev, Alexey A. Dmitriev, Asiya F. Sadritdinova, Irina Y. Karpova, Kirill M. Nyushko, Dmitry V. Kalinin, Nadezhda N. Volchenko, Nataliya V. Melnikova, Kseniya M. Klimina, Dmitry V. Sidorov, Anatoly Y. Popov, Tatiana V. Nasedkina, Andrey D. Kaprin, Boris Y. Alekseev, George S. Krasnov, Anastasiya V. Snezhkina

**Affiliations:** 1 Engelhardt Institute of Molecular Biology, Russian Academy of Sciences, Moscow, Russia; 2 National Medical Research Radiological Center, Ministry of Health of the Russian Federation, Moscow, Russia; 3 Insilico Medicine, Inc., Emerging Technology Centers, Johns Hopkins University Eastern Campus, Baltimore, Maryland USA; 4 Moscow Institute of Physics and Technology, Dolgoprudny, Russia; 5 N.N. Blokhin Russian Cancer Research Center, Moscow, Russia; 6 A.V. Vishnevsky Institute of Surgery, Moscow, Russia; 7 Vavilov Institute of General Genetics, Russian Academy of Sciences, Moscow, Russia; 8 State Hospital №57, Moscow, Russia

**Keywords:** Warburg effect, Hexokinases, shRNA, Glycolysis, Melanoma, Colorectal cancer

## Abstract

**Background:**

The switch from oxidative phosphorylation to glycolysis in proliferating cancer cells, even under aerobic conditions, has been shown first in 1926 by Otto Warburg. Today this phenomenon is known as the “Warburg effect” and recognized as a hallmark of cancer. The metabolic shift to glycolysis is associated with the alterations in signaling pathways involved in energy metabolism, including glucose uptake and fermentation, and regulation of mitochondrial functions. Hexokinases (HKs), which catalyze the first step of glycolysis, have been identified to play a role in tumorigenesis of human colorectal cancer (CRC) and melanoma. However, the mechanism of action of HKs in the promotion of tumor growth remains unclear.

**Results:**

The purpose of the present study was to investigate the effect of silencing of hexokinase genes (*HK1*, *HK2*, and *HK3*) in colorectal cancer (HT-29, SW 480, HCT-15, RKO, and HCT 116) and melanoma (MDA-MB-435S and SK-MEL-28) cell lines using short hairpin RNA (shRNA) lentiviral vectors. shRNA lentiviral plasmid vectors pLSLP-HK1, pLSLP-HK2, and pLSLP-HK3 were constructed and then transfected separately or co-transfected into the cells. *HK2* inactivation was associated with increased expression of HK1 in colorectal cancer cell lines pointing to the compensation effect. Simultaneous attenuation of HK1 and HK2 levels led to decreased cell viability. Co-transfection with shRNA vectors against *HK1*, *HK2*, and *HK3* mRNAs resulted in a rapid cell death via apoptosis.

**Conclusions:**

We have demonstrated that simultaneous inactivation of *HK1* and *HK2* was sufficient to decrease proliferation and viability of melanoma and colorectal cancer cells. Our results suggest that HK1 and HK2 could be the key therapeutic targets for reducing aerobic glycolysis in examined cancers.

## Background

In the beginning of the 20th century, Otto Warburg with his colleagues observed that cancer cells used glycolysis and produced lactate instead of mitochondrial respiration, even in the presence of oxygen and could die through hypoxia if glucose is lacking. Nowadays, this phenomenon is known as “Warburg effect” [1, 2]. Many cancers are characterized by increased aerobic glycolysis [2–5]. In the hypoxic microenvironment, it confers several advantages to cancer cells. Firstly, high rate of glycolysis provides sufficient ATP for tumor cells under reduced mitochondrial function [6–8]. Secondly, glycolysis is a source of the metabolic intermediates (e.g., ribose sugars, glycerol, citrate, nonessential amino acids and NADPH) that are needed for biosynthetic pathways [9]. Finally, tumor cells produce large amount of lactic acid during glucose metabolism that promotes activation of metalloproteinases and matrix remodeling enzymes involved in invasion and metastasis [10]. So, the Warburg effect benefits for the adaptation, proliferation and survival of cancer cells.

Hexokinases (HKs) catalyze the crucial step in glycolysis in which the glucose is phosphorylated to produce glucose-6-phosphate [11]. Four isozymes of hexokinase were found in mammalian tissues: HK1, HK2, HK3 and HK4 (glucokinase) [12, 13]. The alterations in the expression of hexokinase isoenzymes play a role in the tumor initiation and promotion. It has been observed that the tumor cells adapted metabolically primarily by increasing the expression of HK2 [14, 15]. The elevated expression of HK1 was also detected in several tumors, but at lower extent compared to the HK2 isozyme [16–18]. The increased expression of HK3 was shown in colorectal, lung, gastrointestinal, and breast cancers [11, 19]. For liver tumors, a shift in expression from HK4 to HK1 and HK2 was observed [11, 20]. In has been shown that in tumor cells cytosolic HK1 and HK2 were tightly associated to the voltage-dependent anion channel (VDAC) in the mitochondrial membrane [15, 21]. Its interaction has dual function: (1) prevention of mitochondrial outer membrane permeabilization and evasion of subsequent apoptosis, and (2) inhibition of VDAC to facilitate shuttling of ATP from mitochondria into the cytosol [22, 23]. This is also the evidence that HK1 and HK2 are responsible for the accelerated glucose flux in tumor cells. Thus, altered expression of HKs in tumors is a potential target for cancer therapy.

Colorectal cancer (CRC) and malignant melanoma (MM) are very aggressive and deadly cancers with high metastatic rates [24]. The risk of both tumors increases with age [25–28]. Most cases of CRC and melanoma are sporadic and driven by genetic and epigenetic alterations involved in the activation of oncogenes and inactivation of tumor suppressor genes [29–32]. However, around 10-30% of all CRC and 3-15% of MM cases have a hereditary nature [33–35]. CRC and melanoma usually develop without any symptoms for a long time. Many cases of CRC and MM are diagnosed in advanced stages [36–38]. At present, there are few treatment options for patients with CRC or melanoma, but the classical therapies have limited efficiency whereas global incidence of the diseases is increasing very fast [39, 40]. It is important to uncover the molecular mechanisms of the development and progression of CRC and MM for better prevention, diagnosis, and clinical management.

In the present study, to understand the mechanism of aerobic glycolysis in CRC and MM, we investigated the effect of silencing of hexokinase genes in colorectal cancer and melanoma cells using short hairpin RNA (shRNA) lentiviral vectors. Our results suggest HK1 and HK2 as key enzymes for glucose metabolism associated with survival of tumor cells. We determined the significance of HK gene expression in colorectal cancer and melanoma cells and proposed a promising strategy for therapy of the diseases.

## Methods

### Cell cultures

Colorectal adenocarcinoma (HT-29, SW 480, HCT-15, RKO, and HCT 116) and melanoma (MDA-MB-435S and SK-MEL-28) cells were obtained from N.N. Blokhin Russian Cancer Research Center (Moscow, Russia). They were maintained in Dulbecco's modified Eagle's medium (DMEM) (Thermo Fisher Scientific, USA) supplemented with 10% FBS (Harlan Sera-Lab, UK), penicillin (100 U/ml), and streptomycin (100 μg/ml) (Thermo Fisher Scientific, USA). The cells were cultured at 37 °C in a 5% CO_2_ atmosphere and passaged every 2–3 days by dissociation with trypsin (Thermo Fisher Scientific, USA).

### Constructs and production of lentivirus

Nine hairpin RNAs were constructed to specifically target *HK1*, *HK2,* and *HK3* mRNA (3 interference sequences per each gene) by shRNA design tools (http://rnaidesigner.thermofisher.com/rnaiexpress/). Using BLAST (http://blast.ncbi.nlm.nih.gov/Blast.cgi) we have seen that the designed shRNAs targeted only the selected genes. ShRNAs were synthesized by Evrogen (Russia) (Table 1). Their sequences were annealed and cloned between EcoRI and BamHI restriction sites in the pLSLP lentiviral vector and checked by Sanger sequencing on ABI Prism 3100 Genetic Analyzer (Thermo Fisher Scientific, USA).

**Table 1:** shRNA sequences

Primer name	shRNA sequences
HK1_sh1_t	gatccgGGAACTGAGGCACATTGATCTCACGTGAGATCAATGTGCCTCAGTTCCtttttg
HK1_sh1_b	aattcaaaaaGGAACTGAGGCACATTGATCTCACGTGAGATCAATGTGCCTCAGTTCCcg
HK1_sh2_t	gatccgGCCTTTGGAGACGATGGATCACACGTGTGATCCATCGTCTCCAAAGGCtttttg
HK1_sh2_b	aattcaaaaaGCCTTTGGAGACGATGGATCACACGTGTGATCCATCGTCTCCAAAGGCcg
HK1_sh3_t	gatccgGGAAGCAGACGCACAACAATGCACGTGCATTGTTGTGCGTCTGCTTCCtttttg
HK1_sh3_b	aattcaaaaaGGAAGCAGACGCACAACAATGCACGTGCATTGTTGTGCGTCTGCTTCCcg
HK2_sh1_t	gatccgGGGTGAAAGTAACGGACAATGCACGTGCATTGTCCGTTACTTTCACCCtttttg
HK2_sh1_b	aattcaaaaaGGGTGAAAGTAACGGACAATGCACGTGCATTGTCCGTTACTTTCACCCcg
HK2_sh2_t	gatccgGCAGAAGGTTGACCAGTATCTCACGTGAGATACTGGTCAACCTTCTGCtttttg
HK2_sh2_b	aattcaaaaaGCAGAAGGTTGACCAGTATCTCACGTGAGATACTGGTCAACCTTCTGCcg
HK2_sh3_t	gatccgGGGACTTTGATATCGACATTGCACGTGCAATGTCGATATCAAAGTCCCtttttg
HK2_sh3_b	aattcaaaaaGGGACTTTGATATCGACATTGCACGTGCAATGTCGATATCAAAGTCCCcg
HK3_sh1_t	gatccgGGGTGACTCTAACTGGCATTGCACGTGCAATGCCAGTTAGAGTCACCCtttttg
HK3_sh1_b	aattcaaaaaGGGTGACTCTAACTGGCATTGCACGTGCAATGCCAGTTAGAGTCACCCcg
HK3_sh2_t	gatccgGCTGAATGTGGTTGCCATTGTCACGTGACAATGGCAACCACATTCAGCtttttg
HK3_sh2_b	aattcaaaaaGCTGAATGTGGTTGCCATTGTCACGTGACAATGGCAACCACATTCAGCcg
HK3_sh3_t	gatccgGGCTTCGGATGTTGAGCTTGTCACGTGACAAGCTCAACATCCGAAGCCtttttg
HK3_sh3_b	aattcaaaaaGGCTTCGGATGTTGAGCTTGTCACGTGACAAGCTCAACATCCGAAGCCcg

### Cell transfection

The constructed vectors were transfected into the cells using Lipofectamin 2000 (Thermo Fisher Scientific, USA) according to manufacturer's instructions. The transfected cells were then selected by puromycin (10 mg/ml) for 5 days. The puromycin-resistant colonies were then picked and expanded. HK expression levels in selected clones were determined by quantitative PCR (qPCR) and Western blot analysis.

### RNA extraction and cDNA synthesis

The total RNA was isolated using RNeasy Mini kit (Qiagen, Germany) according to manufacturer's protocol. RNA quality was monitored with absorbance spectra (NanoDrop Technologies Inc., USA) and the RNA integrity number (RIN; Agilent Technologies, USA). The RNA samples were treated with DNase I (Thermo Fisher Scientific, USA). cDNA was synthesized using M-MLV Reverse Transcriptase (Thermo Fisher Scientific, USA) and random primers.

### Quantification of gene expression by qPCR

Gene expression was analyzed using qPCR with TaqMan Gene Expression Assays (Thermo Fisher Scientific, USA) and TaqMan Universal Master Mix (Thermo Fisher Scientific, USA) according to the manufacturer’s recommendations. The reactions were performed using AB 7500 Real-Time PCR System (Thermo Fisher Scientific, USA) with RQ (Relative Quantitation) software (Thermo Fisher Scientific, USA). PCR program was as follow: 10 min at 95 °C, then 50 two-step cycles 15 s at 95 °C and 60 s at 60 °C. Total reaction volume was 20 μl in triplicate. PCR products were analyzed in 2% agarose gels and nucleotide sequences of the amplicons were verified by Sanger sequencing on ABI Prism 3100 Genetic Analyzer (Thermo Fisher Scientific, USA).

### Western blot analysis

Proteins were extracted using radioimmunoprecipitation assay lysis buffer (RIPA buffer). The concentration and purity of proteins were analyzed on Agilent Bioanalyzer 2100 (Agilent Technologies, USA). Subsequently, proteins (20 μg) were separated by electrophoresis on a SDS-PAGE gel and transferred onto a polyvinylidene fluoride membrane. The membrane was blocked in 3% BSA in Tris-buffered saline in 0.1% Tween 20 (TBST) at room temperature for 1 h and then incubated overnight in the primary antibody solution (MA5-14789, MA5-14849, and PA5-29304) (Thermo Fisher Scientific, USA) at 4 °C. Next, the samples were washed three times with TBST, and the membrane was incubated with secondary antibody solution (A-11008) (Thermo Fisher Scientific, USA) for 1 h at room temperature. After incubation, the samples were washed five times and the bands were detected using Pierce ECL Western Blotting Substrate (Thermo Fisher Scientific, USA) on a Gel Doc XR+ System (Bio-Rad, USA) and analyzed with Image Lab Software (Bio-Rad, USA). β-actin ﻿was used for normalization.

### Cell proliferation and viability assay

Cell proliferation and viability were measured by MTT assay (Promega, USA) according to the manufacturer’s instructions. The transfected cells were incubated with tetrazolium dye solution. The formazan absorbance was then recorded at 570 nm using a multi-mode microplate reader CHAMELEON V (Hidex, Turkey).

### Analysis of DNA fragmentation

The apoptosis in transfected cells was determined using Apoptotic DNA-Ladder Kit (Roche, Switzerland) according to the manufacturer’s protocol. The DNA samples were electrophoresed on a 2% agarose gels containing 1 μL/10 mL GelGreen nucleic acid gel stain (Biotium, USA). The gels were examined by an ultraviolet gel documentation system Gel Doc XR+ System (Bio-Rad, USA). Apoptosis was visualized as a ladder pattern of 180–200 bp due to DNA cleavage by the activation of a nuclear endonuclease.

### Statistical analysis

We applied the nonparametric Wilcoxon/Mann–Whitney U–test to measure statistical significance between the two groups. We used Kruskal-Wallis test for multiple comparisons. Data were considered significant at *P* < 0.05. The statistical procedures were performed with a BioStat 2009 professional software (AnalystSoft Inc., USA).

## Results

### Expression of HK mRNA in cell lines is silenced by shRNA

**Fig. 1:**
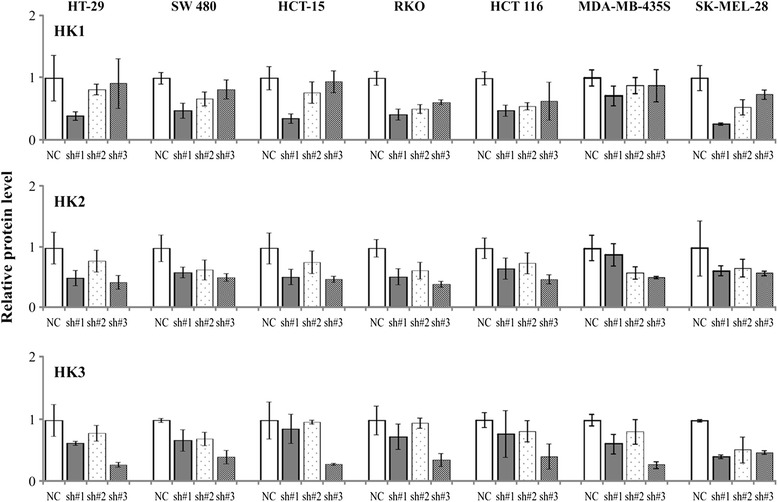
The expression levels of HK1, HK2, and HK3 in negative control (NC) and shRNA transfected cells

In order to silence the expression of HK mRNA in colorectal cancer and melanoma cells, the cell lines were transfected with HK1, HK2, and HK3 shRNA (three shRNAs per each gene: sh#1, sh#2, and sh#3). qPCR and Western blot analysis showed that HK1 sh#1, HK2 sh#3, and HK3 sh#3 significantly repressed the expression of target genes in cells to be tested (Fig. 1). Therefore, these shRNAs were used in the following experiments.

### HK mRNA expression levels in cells transfected separately with three lentiviral vectors pLSLP-HK1, pLSLP-HK2, and pLSLP-HK3

**Fig. 2:**
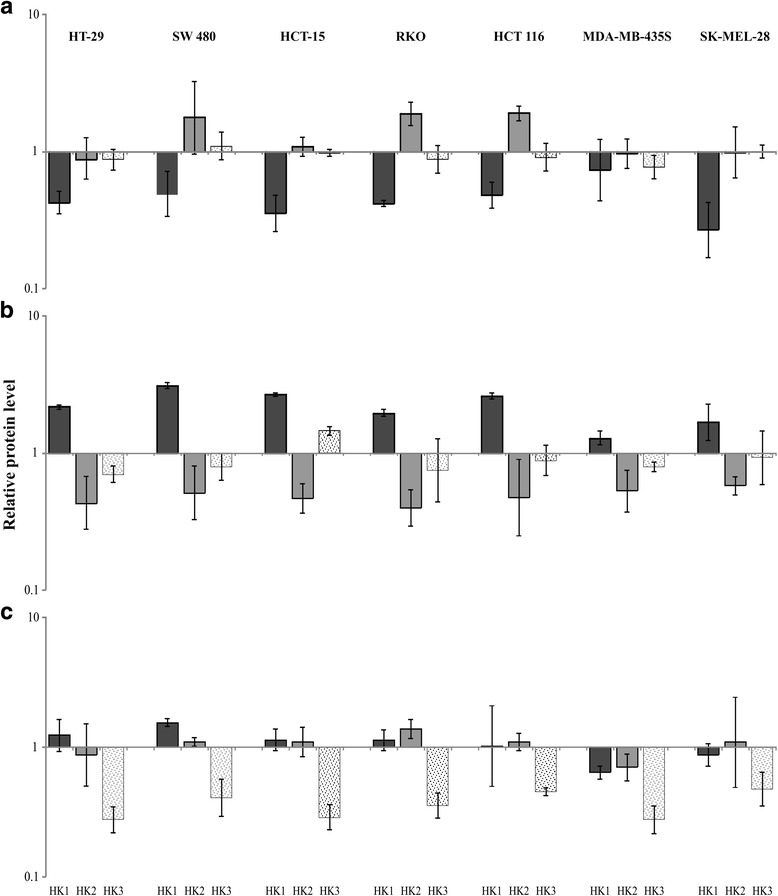
The relative protein level of three genes (*HK1*, *HK2*, and *HK3*) in colorectal cancer (HT-29, SW 480, HCT-15, RKO, and HCT 116) and melanoma (MDA-MB-435S and SK-MEL-28) cell lines with **a** *HK1*, **b** *HK2*, and **c** *HK3* knockdown

Expression levels of HKs in cell lines that were separately transfected with three lentiviral vectors against *HK1*, *HK2*, and *HK3* genes were determined using qPCR and Western blot analysis. The expression levels of HK1 and HK2 were not significantly changed in both colorectal cancer and melanoma cells with HK3 knockdown. We observed increased expression of HK1 in colorectal cancer cells transfected by vector pLSLP-HK2, but not in melanoma ones; the mRNA and protein levels of HK3 were not altered in all cases. Expression differences of HK2 and HK3 between pLSLP-HK1 transfected cells and control were not significant. Generally, we showed the absence of compensatory expression between the *HK* genes in melanoma cells. These data suggest that the increased expression of *HK1* gene may play a role in maintaining of high rates of glycolysis in colorectal cancer cells when *HK2* is suppressed (Fig. 2).

### Simultaneous down-regulation of HK expression induces apoptosis and inhibits tumor growth in vitro

**Fig. 3:**
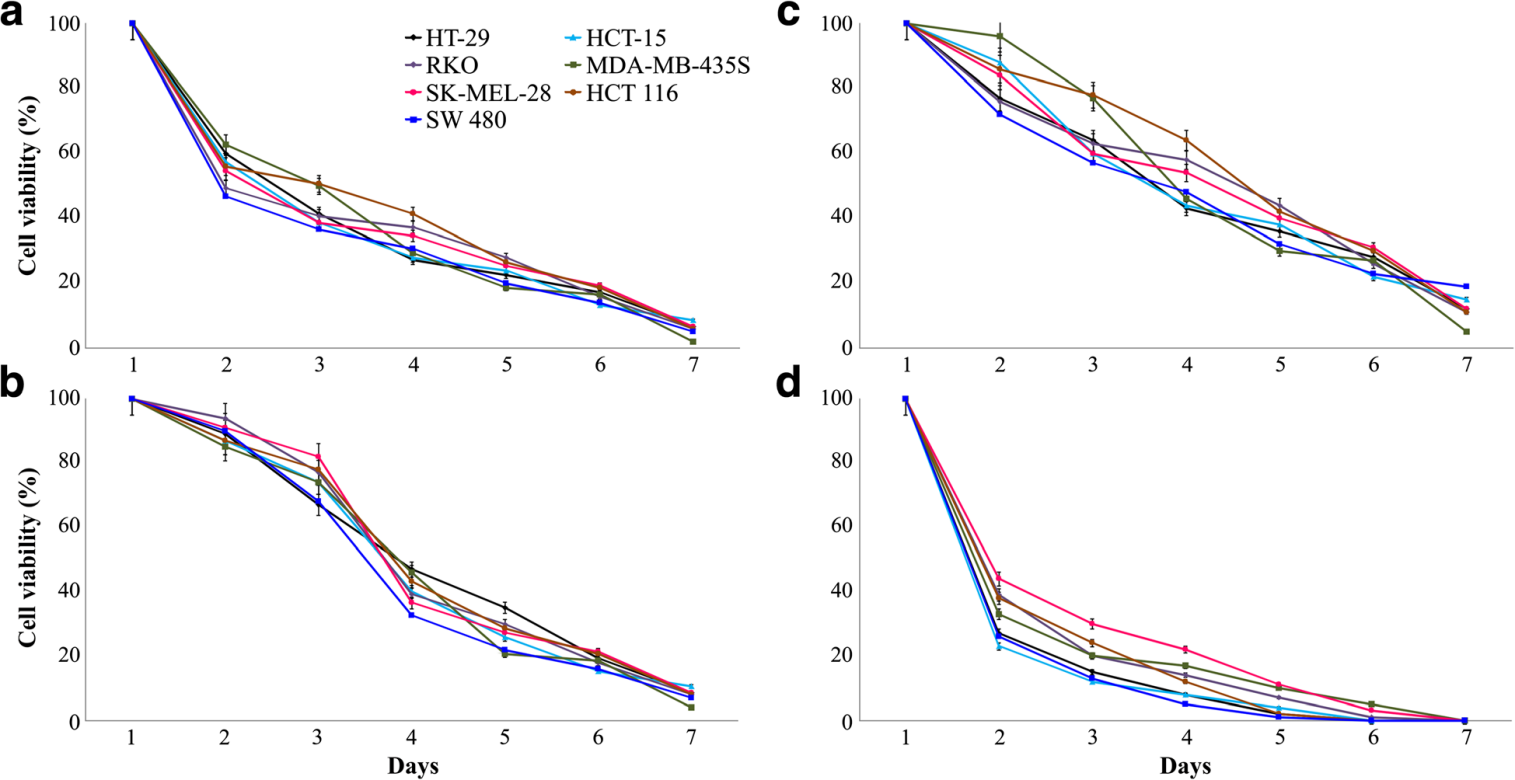
Inhibitory effect of *HK1*, *HK2* and *HK3* knockdown on cell growth and proliferation. Viability of colorectal cancer HT-29 (black), SW 480 (blue), HCT-15 (light blue), RKO (purple), HCT 116 (brown) and melanoma MDA-MB-435S (green), SK-MEL-28 (red) cell lines transfected by lentiviral vectors pLSLP-HK1 plus pLSLP-HK2 (**a**), pLSLP-HK1 plus pLSLP-HK3 (**b**), pLSLP-HK2 plus pLSLP-HK3 (**c**), and pLSLP-HK1, pLSLP-HK2 plus pLSLP-HK3 (**d**)

As shown in Fig. 3, cells transfected by different combinations of lentiviral vectors against hexokinases showed decreased viability and time-dependent inhibition of proliferation. This effect is stronger in cells with simultaneous knockdown of *HK1*, *HK2* and *HK3* genes. Both colorectal cancer and melanoma cells are more sensitive to *HK1* and *HK2* deficiency. Viability of the cells transfected by lentiviral vectors pLSLP-HK1 and pLSLP-HK2 was lower than cells transfected by other double combination of ones (pLSLP-HK1 and pLSLP-HK3 or pLSLP-HK2 and pLSLP-HK3).

Formation of fragmented DNA is one of the typical apoptotic features. We performed DNA fragmentation assay to reveal whether apoptosis plays an important role in cell death. Multiple DNA fragments were detected in the cells co-transfected by pLSLP-HK1, pLSLP-HK2, and pLSLP-HK3. These data suggest that simultaneous down-regulation of *HK1*, *HK2*, and *HK3* gene expression could induce apoptosis in colorectal cancer and melanoma cells.

## Discussion

Activation of aerobic glycolysis occurs in almost all cancer cells. The process has a very strong regulatory system, because in addition to ATP production glycolysis supplies actively proliferating tumor cells with building blocks [41, 42]. Hexokinases, as the key glycolytic enzymes, may be regulated more extensively in glycolysis process [43]. We have previously shown deregulation in the expression of *HK* genes in colorectal cancer [19]. In this study, using shRNA-based gene knockdown we have checked the compensatory expression between the HK genes, and analyzed the viability of colorectal cancer and melanoma cells when various hexokinase isoenzymes were inactive. We have shown that shRNA-mediated attenuation of *HK1* and *HK2* together led to decreased cell viability. *HK2* gene inactivation was associated with increased expression of HK1 in colorectal cancer cells. The compensatory expression between the *HK* genes was not detected in melanoma cells. Co-transfection by shRNA vectors against mRNA of *HK1*, *HK2*, and *HK3* genes resulted in a rapid cell death by apoptosis.

HK1 and HK2 play an important role in glycolysis [41]. They are associated with the outer mitochondrial membrane *via* VDAC and implicated in cell survival [13, 44–49]. *HK2* expression is limited in most normal tissues, but frequently up-regulated in cancer [48, 50–52]. It is known that *HK2* is a target for several oncogenic transcription factors (HIF-1, Myc, and p53) [42], and is involved in Akt signaling pathway [43]. The overexpression of HK2 provides tumor cells with a growth advantage due to increased glycolytic activity, prevents from apoptosis, and increases their possibility for metastasis [53]. High HK2 expression in lung, ovarian, pancreatic, breast cancers and hepatocellular carcinoma was shown to be associated with poor patient prognosis [50, 54–58].

Bryson and colleagues have demonstrated that primary increase in HK1 activity reduced susceptibility of renal epithelial cells to oxidant-induced cell death [59]. The series of studies have shown up-regulation of HK1 in several tumors, including colorectal, gastric, and thyroid cancer, and supposed it as an unfavorable prognostic factor [60–62]. We observed increased expression of HK1 in colorectal cancer cells with *HK2* gene silencing, but not in melanoma cells. In cells with *HK1* or *HK3* knockdown, change in HK1 expression was insignificant. Simultaneous down-regulation of *HK1* and *HK2* genes led to reduction of cell proliferation and viability compared to double knockdown of *HK1*/*HK3* or *HK2*/*HK3* genes. Noteworthy, Patra et al. have demonstrated that oncogenic HK2 expression and activity cannot be compensated by HK1 in mouse embryonic fibroblasts [54]. Our results confirm that HK1 and HK2 are involved in tumor growth maintenance. However, we can assume that despite the increase in the expression of HK1 in colorectal cancer it may be insufficient to maintain high level of aerobic glycolysis. Overexpression of HK1 in tumors seems to be the mechanism for the protection of cancer cells against oxidative stress and apoptosis, as well.

HK3 activity is regulated by HIF-dependent pathway and glucose level. Overexpression of HK3 results in increased cellular ATP and reduced ROS production, and promotes the expression of genes involved in mitochondrial biogenesis. These processes can mediate the cytoprotective effect of HK3 [43]. In the study, the expression levels of HK1 and HK2 were not significantly changed in cells with HK3 knockdown that indicate its lower importance in the regulation of glycolysis rate.

## Conclusion

We have demonstrated that simultaneous HK1 and HK2 deficiency results in decreased cell survival whereas inactivation of HK1, HK2, and HK3 led to rapid cell death *via* apoptosis. Inactivation of HK2 was followed with up-﻿regulation of HK1 expression in colorectal cancer, but not in melanoma cells. Taken together, our results suggest *HK1* and *HK2* genes as the potential molecular targets for colorectal cancer and melanoma therapy.
